# Millennials Versus Boomers: An Asymmetric Pattern of Realistic and Symbolic Threats Drives Intergenerational Tensions in the United States

**DOI:** 10.1177/01461672231164203

**Published:** 2023-05-03

**Authors:** Stéphane P. Francioli, Felix Danbold, Michael S. North

**Affiliations:** 1 University of Pennsylvania, Philadelphia, USA; 2 University College London School of Management, UK; 3 New York University, New York City, USA

**Keywords:** intergroup threat theory, entitativity, ageism, generation

## Abstract

Intergenerational conflict appears frequently in American public discourse, often framed as clashes between Millennials and Baby Boomers. Building on intergroup threat theory in an exploratory survey, a preregistered correlational study, and a preregistered intervention (*N* = 1,714), we find that (a) Millennials and Baby Boomers do express more animosity toward each other than toward other generations (Studies 1–3); (b) their animosity reflects asymmetric generational concerns: Baby Boomers primarily fear that Millennials threaten traditional American values (symbolic threat) while Millennials primarily fear that Baby Boomers’s delayed transmission of power hampers their life prospects (realistic threat; Studies 2–3); (c) finally, an intervention challenging the entitativity of generational categories alleviates perceived threats and hostility for both generations (Study 3). These findings inform research on intergroup threat, provide a theoretically grounded framework to understand intergenerational relations, and put forward a strategy to increase harmony in aging societies.

In the increasingly age-diverse United States (North & Fiske, 2012), generational identities have become a popular subject of conversation. One central narrative in these discussions is the purported rift between *Baby Boomers* (people born between 1946 and 1964) and *Millennials* (people born between 1981 and 1996), the two largest (Pew Research Center, 2020) and most talked about (Google Trends, 2021) adult generations in the United States.^1^ Millennials are often depicted by their elders as lazy, entitled, disrespectful, and responsible for the perceived decay of the American way of life (Paul, 2017). On the contrary, Baby Boomers also come under fire, portrayed by younger critics as greedy, complacent, wasteful, and taking advantage of economic, environmental, and political resources at the expense of other generations (e.g., Lopez, 2016; Romano, 2019).

Contrasting with these popular narratives, scholars have challenged the assertion that generational affiliation predicts meaningful differences in personality, values, or worldview, stressing instead that perceived dissimilarities are best explained by situational factors and life stage differences (Aboim & Vasconcelos, 2014; Costanza et al., 2012; National Academies of Sciences, Engineering, and Medicine, 2020; Parry & Urwin, 2011; Rudolph et al., 2021). However, the widespread use of generational labels in mainstream media, national polls, online forums, political speeches, and corporate trainings indicates that generational categories might function as powerful social identities (e.g., Tajfel & Turner, 1979) and catalysts of meaningful intergroup relations (e.g., North & Shakeri, 2019; Timonen & Conlon, 2015).

Generational identities differ markedly from more commonly studied age-based identities (e.g., younger and older adults). Age categories have no clear boundaries, (i.e., they may be idiosyncratically defined by each individual) and are transitory (i.e., today’s old were once young, and today’s young will one day be old). In contrast, generational categories are rigid, collectively defined, and fixed across the lifespan. As such, generations differ from age-based identities in that they are highly entitative (i.e., they represent clearly defined groups whose members are seen as a coherent and homogeneous whole; Agadullina & Lovakov, 2018; Campbell, 1958). Prior work has shown that group entitativity fosters an us-versus-them mentality that intensifies intergroup conflicts (Effron & Knowles, 2015). Given the entitativity of generations, we propose that intergenerational tensions—such as those observed between Millennials and Baby Boomers—constitute a domain of intergroup relations ripe for social psychological study.

Mirroring popular intergenerational narratives, we predict and find that Millennials and Baby Boomers harbor more hostile attitudes toward each other than toward any other generation. To explain this mutual animosity, we build upon intergroup threat theory (ITT; Stephan et al., 2009; Stephan & Stephan, 2000), to predict that intergenerational tensions are rooted in a unique pattern of realistic and symbolic threat. Whereas prior ITT findings suggest that dominant groups’ animosity toward outgroup members is driven primarily by realistic threat (i.e., perceived conflict over economic opportunities and power; Rios et al., 2018), we find that the resentment of Baby Boomers (economically and politically dominant) toward Millennials (the nondominant group) is driven primarily by symbolic concerns (i.e., perceived conflict over culture, values, and worldview). In contrast, Millennials’s resentment toward Baby Boomers is driven primarily by practical concerns over their life prospects (i.e., realistic threat).

Identifying the causes of intergenerational tensions also begs the question of how these tensions may be alleviated. The threat asymmetry we predict between the two generations suggests that it might be difficult to develop a “one-size-fits-all” threat intervention that would directly attend to both Millennials’s realistic concerns and Baby Boomers’s symbolic concerns simultaneously. Furthermore, prior work has shown that trying to challenge perceptions of intergroup threats can elicit reactance (Rios et al., 2018). To circumvent these issues, we test an alternative intervention in which we challenge the entitativity of generations and promote instead the more fluid, unifying, and universal experience of age and aging. In doing so, we aim to reduce both perceived threat and outgroup animosity by weakening the very foundation upon which these threats and animosity lie.

Taken together, this work advances scholarship on intergroup conflicts and intergroup threat, provides a theoretically grounded framework to understand a major rift in contemporary U.S. society, and offers a potential strategy to reduce intergenerational tensions.

## Intergroup Threats Between Generations

ITT posits that tensions between groups result from both perceived concerns over the ingroup’s power, status, resources, and well-being (i.e., realistic threats), and fears for the ingroup’s culture, values, worldview, and way of life (i.e., symbolic threats; Stephan et al., 2009). Although outgroups generally elicit a mix of both realistic and symbolic threats, prior research suggests that the relative societal standing of the ingroup shapes the extent to which either threat drives intergroup bias (e.g., Morrison et al., 2009; Riek et al., 2006; Rios et al., 2018; Stephan et al., 2009). Therefore, understanding the societal standing of Baby Boomers and Millennials relative to one another may help predict the nature of the threat driving each generation’s animosity toward the other.

On many metrics, Baby Boomers constitute the dominant group among current U.S. generations. Although research has shown that people tend to stereotype older adults as vulnerable and lacking in both competence and agency (Cuddy et al., 2005; Cuddy & Fiske, 2002; Kite et al., 2005), these assumptions map onto the broad category of “older people,” which conflates “younger” older adults (i.e., Baby Boomers) with their older counterparts: the Silent Generation. Occupying the younger range of older adulthood, Baby Boomers have benefited from advances in medicine, working conditions, and standards of living that may exempt them from many of the stereotypes associated with advanced aging. Furthermore, Baby Boomers occupy a clear position of economic, social, and political dominance in contemporary America. They are on average about 10 times wealthier than Millennials (Hoffower, 2020); they are overrepresented in positions of economic power (e.g., CEOs of Fortune 500; Udland, 2019); they form a major and highly reliable voting bloc; and they dominate both state and national politics (e.g., 65% of the U.S. Senate, 48% of the House of Representatives, and 68% of governorships). In contrast, Millennials fall far below Baby Boomers in their access to material resources and political influence. Illustrative of this lower societal standing, Millennials have a lower rate of home ownership than prior generations at the same age (Leonhardt, 2021) and the lowest political representation in the federal government (Pew Research Center, 2021a). Thus, potential intergenerational conflicts opposing Baby Boomers and Millennials see the former generation as the dominant group and the latter as the nondominant one.

### Symbolic Threat Drives Baby Boomers’s Bias Against Millennials

Given Baby Boomers’s dominant standing, prior ITT findings suggest that realistic concerns might drive their hostility toward Millennials, as they have “the most to lose” (Rios et al., 2018, p. 237). In support of this idea, many Baby Boomers have expressed the desire to remain in the workforce longer and some see Millennials—viewed by employers as cheaper and more adaptable—as standing in their way (Francioli & North, 2021a; Kulik et al., 2016; Loretto & White, 2006). Furthermore, the societal standing of generations is inherently dynamic, and societal norms often compel older generations to relinquish their advantages to younger generations. Nonetheless, older generations still tend to enjoy the comfort and wealth they accumulated over time. As such, Baby Boomers may not see Millennials as a credible resource threat, particularly in their lifetime. Hence, we do not expect realistic concerns to drive Baby Boomers’s hostility toward Millennials.

On the contrary, Baby Boomers may be more concerned with the cultural legacy they leave behind. Older generations often expect younger ones to respect, honor, and preserve their way of life (Cruz-Saco, 2010), a desire potentially amplified as one’s generation approaches an unavoidable numerical decline (Danbold & Huo, 2015, 2022). In a context where Millennials have long been depicted as challenging the norms and values of previous generations (Paul, 2017), Baby Boomers may see Millennials as a threat to the cultural imprint they wish to leave on American society. This may be especially true among (older) White Americans, most attached to the traditional American values that Millennials are perceived to challenge (Danbold & Huo, 2022). For this reason, we predict that symbolic concerns will constitute the primary driver of Baby Boomers’s hostility toward Millennials.

### Realistic Threat Drives Millennials’s Bias Against Baby Boomers

Whether Millennials’s hostility toward Baby Boomers is anchored in realistic or symbolic threat is more of an open question. The ITT literature is less consistent as to which threat drives the antagonism of nondominant groups toward dominant ones. Some evidence suggests that symbolic threat might be the strongest predictor of Millennials’s bias. For instance, young people often frown upon older adults who adopt elements of youth culture (e.g., going into night clubs, using social media; North & Fiske, 2013). They may also be frustrated by Baby Boomers’s critiques of their cultural contributions (e.g., Millennials’s values, political views, lingo, and technology adoption). Nonetheless, Millennials may recognize that the norms and values of younger generations typically become mainstream over their lifespan (Gilleard, 2004; Mannheim, 1928; Ryder Norman, 1965), particularly as older generations decline numerically. As such, we do not expect symbolic concerns to drive Millennials’s antagonism toward Baby Boomers.

Instead, we expect Millennials to be most concerned by their own economic situation and life prospects. As Baby Boomers live longer, delay retirement, and retain powerful societal roles, Millennials may worry that Baby Boomers’s hold over economic and political resources threatens their own opportunity to accumulate wealth, resources, and power. These concerns may be exacerbated by economic setbacks that have saddled many Millennials with vast debts and rising expenses, hindering their ability to establish themselves and live prosperously (Resolution Foundation, 2018; Van Dam, 2020). In this context, Baby Boomers’s deferred withdrawal from positions of influence may be seen as a jam in the natural order of generational transmission of power and wealth (North & Fiske, 2013). As such, we predict that realistic threat constitutes the primary driver of Millennials’s hostility toward Baby Boomers.

## Reducing Intergroup Tensions by Reducing Generational Entitativity

If intergroup threats drive intergenerational conflicts, it is valuable to explore how these threats—and ultimately, these tensions—may be reduced. Although social psychologists often try to attenuate an unfavorable outcome (e.g., negative intergroup attitudes) by targeting its mechanism (e.g., intergroup threat), improving intergenerational tensions via a direct threat intervention might prove challenging. First, the threat asymmetry we predict for Millennials and Baby Boomers means that each generation would require a message tailored to their own unique outgroup anxiety (i.e., realistic threat for Millennials and symbolic threat for Baby Boomers). Second, prior work suggests that interventions aimed directly at reducing intergroup threat are not always effective, as people generally reject direct claims that their ingroup’s concerns are unfounded (Rios et al., 2018). Although we do not argue that it is impossible to assuage the concerns of both generations via messages directly targeting their unique concerns, we propose and test a potentially more efficient, indirect solution to alleviate both Millennials’s and Baby Boomers’s experience of threats in a single intervention.

Research suggests that intergroup prejudice is exacerbated when people perceive the ingroup and outgroup to be clearly and legitimately defined, such that members of each group form a coherent, homogeneous whole (i.e., entitativity; Agadullina & Lovakov, 2018; Campbell, 1958). Evidence also suggests that perceived group entitativity can be manipulated. For example, Effron and Knowles (2015) successfully reduced outgroup bias by challenging the entitativity of people’s ingroup. Elaborating on this idea, we tested whether challenging the foundation of generational identities alleviates feelings of intergenerational threat—and thus, reduces outgroup prejudice—by blurring the lines between ingroups and outgroups and by calling into question the social identities upon which these feelings of threat are based. This strategy may be particularly well suited to generational groups, given that many scholars are already critical of their legitimacy (Costanza et al., 2012; National Academies of Sciences, Engineering, and Medicine, 2020; Parry & Urwin, 2011; Rudolph et al., 2021). Furthermore, it is also possible to weaken the entitativity of generational groups by increasing the salience of age (Weiss & Lang, 2012). Contrary to generational identities, which are fixed across the life span, age- or life-stage-based identities are fluid, universal, and unifying (i.e., we all pass through each age group, inhibiting ingroup vs. outgroup distinctions). As such, we predict that an intervention challenging the legitimacy and entitativity of generational categories and encouraging a shift toward life stage thinking may effectively reduce the perceived entitativity of all generational identities (ingroup and outgroups), lessening the extent to which both Millennials and Baby Boomers see each other as a source of threat and antagonism.

## Research Overview

We test our predictions across three studies. First, we examine Baby Boomers’s and Millennials’s attitudes toward different generations to determine whether the popular “Boomer-versus-Millennial” narrative reflects real-world tensions (Studies 1–3). Second, to better define the nature of these tensions, we test whether realistic concerns drive Millennials’s perceptions of Baby Boomers and symbolic concerns Baby Boomers’s perceptions of Millennials (Studies 2–3). Third, we test whether challenging the entitativity of generations helps alleviate these concerns (Study 3). Of note, participants in Studies 1 and 2 were recruited solely based on generational membership and are predominantly White Americans. Study 3 includes a sample more representative of each generation’s gender, racial, and political makeup. We discuss further the implications of the demographic makeup of our samples in the limitation section.

Experiment 1 was not preregistered. The preregistration of the sample size, exclusion criteria, study design, predictions, and planned analyses for Experiments 2 and 3 can be accessed, respectively, here: https://aspredicted.org/XKJ_558 and here: https://aspredicted.org/SW5_HDR. De-identified data for all experiments along with their codebooks and data analysis scripts are posted here: https://osf.io/vm97p/?view_only=0b28ba84a9e04003abf79418192b1a03. The detailed material for these studies is available in the appendix.

## Study 1

In Study 1, we asked Baby Boomers and Millennials how they felt toward each of the four largest U.S. adult generations (i.e., Millennials, Gen-Xers, Baby Boomers, and Silent Generation), and the extent to which each outgroup generation posed a threat to their ingroup. We expected Baby Boomer and Millennial participants to report more animosity and concern toward one another’s generation than toward other generations.

### Methods

#### Participants

We collected 425 complete U.S.-based responses from Amazon Mechanical Turk. Given the exploratory nature of this study, we had no a priori expectations about effect sizes and did not conduct a priori power analyses. Eligible respondents were born between 1981 and 1996 (i.e., Millennials) or 1946 and 1964 (i.e., Baby Boomers). We excluded 21 respondents based on duplicate IP addresses (including these participants into our analyses did not materially change our results). Our final sample included 299 Millennials (age: *M* = 30.4, *SD* = 4.09; 142 women; 29.4% ethnic minorities: 46 African Americans, 18 Asian Americans, 22 Latinos/Hispanic Americans, and two participants whose ethnic background was not listed) and 108 Baby Boomers (age: *M* = 60.0, *SD* = 4.94; 69 women; 8.3% ethnic minorities: seven African Americans and two Latinos/Hispanic Americans), forming a total of 407 participants. Hence, the study comprises a Millennial–Baby Boomer ratio of roughly 3:1, mirroring the broader MTurk pool.^2^

#### Procedure and Measures

Participants completed our primary dependent variables (DVs), followed by a brief demographic questionnaire and debriefing.

##### Attitudes Toward Each Generation

Participants shared their feelings toward the four largest U.S. adult generations (i.e., Millennials, Gen-Xers, Baby Boomers, and Silent Generation) using feeling thermometers with endpoints 0 = “*you feel extremely cold/unfavorable toward that group*,” and 10 = “*you feel extremely warm/favorable toward that group*.” We did not include—the younger—Generation Z as there are still debates about its boundaries and many of its likely members are still children.

##### General Threat

Participants reported the extent to which each outgroup cohort (e.g., Gen-Xers, Baby Boomers, and Silents for Millennials) represented a threat to the interest of their generation using a 100-point scale with endpoints 0 = *Not at All* and 100 = *Extremely*. At this stage, no distinction between realistic or symbolic threat was specified; the measure captured a global sense of threat.

### Results

Descriptive statistics and correlation matrix in Table 1.

**Table 1.** Study 1 Descriptive Statistics and Correlations Between Attitudes and Threat, by Participant and Target Generation

Participant	Target	Attitudes		Threat		Attitudes/threat correlations	
		*M*	*SD*	*M*	*SD*	*r*	*p*
Millennials	Millennials	6.88	2.67				
	Gen-Xers	6.57	2.44	38.7	29.8	−.02	.750
	Baby Boomers	5.61	2.92	45.5	32.2	−.30	<.001
	Silent Generation	6.35	2.83	33.7	31.4	−.20	<.001
Baby Boomers	Millennials	5.27	2.80	41.9	36.5	−.47	<.001
	Gen-Xers	6.57	2.23	33.1	29.1	−.20	.040
	Baby Boomers	7.74	2.12				
	Silent Generation	7.72	2.45	16.4	24.6	−.30	.002

#### Attitudes Toward Each Generation

**Figure 1.**
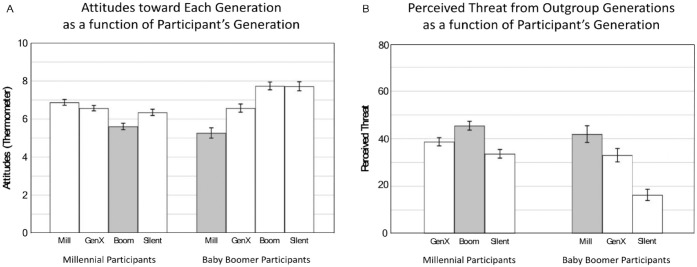
Millennials and Baby Boomers’s Attitudes Toward and Perceived Threats From the Four U.S. Adult Generations. *Note.* Panel A = Attitudes; Panel B = Perceived Outgroup Threat. Gray bars indicate the outgroup of interest (i.e., Millennials for Baby Boomer participants and Baby Boomers for Millennial participants). Error bars indicate ±1 *SE*.

We conducted a mixed two-way analysis of variance (ANOVA) on attitudes with participant generation and target generation as predictors and followed up on a significant two-way interaction, *F*(3, 1,215) = 41.85, *p* < .001, with pairwise comparisons (see Figure 1A). Millennials rated Baby Boomers the least favorably (*M* = 5.61; *SD* = 2.92); below ingroup members (*M* = 6.88; *SD* = 2.67), *p* < .001, *d* = 0.33; Gen-Xers (*M* = 6.57; *SD* = 2.44), *p* < .001, *d* = 0.37; and Silents (*M* = 6.35; *SD* = 2.83), *p* < .001, *d* = 0.27. Conversely, Baby Boomers reported the least favorable attitudes toward Millennials (*M* = 5.26; *SD* = 2.80); below Gen-Xers (*M* = 6.57; *SD* = 2.23), *p* < .001, *d* = 0.45; ingroup members (*M* = 7.74; *SD* = 2.11), *p* < .001, *d* = 0.72; and Silents (*M* = 7.72; *SD* = 2.45), *p* < .001, *d* = 0.64.

#### General Threat

To examine the extent to which each cohort perceived other generations as a threat, we ran two separate one-way repeated ANOVAs (i.e., one for each participant generation) followed by pairwise comparisons (see Figure 1B). Results paralleled those of attitudes. Millennials perceived Baby Boomers (*M* = 45.47/100; *SD* = 32.18) as the most threatening to their generation’s interests; above Gen-Xers (*M* = 38.71; *SD* = 29.75), *p* < .001, *d* = 0.15; and Silents (*M* = 33.66; *SD* = 31.42), *p* < .001, *d* = 0.25; *F*(2, 596) = 25.16. Conversely, Baby Boomers perceived Millennials as the biggest threat (*M* = 41.94; *SD* = 36.43); above Gen-Xers (*M* = 33.06; *SD* = 29.15), *p* = .008, *d* = 0.19; and Silents (*M* = 16.37; *SD* = 24.61), *p* < .001, *d* = 0.65; *F*(2, 214) = 30.71, *p* < .001.

#### General Threat Predicts Attitudes

Consistent with our expectations, intergroup threat negatively predicted attitudes for both Millennials rating Baby Boomers (*r* = −.30, *p* < .001) and Baby Boomers rating Millennials (*r* = −.47, *p* < .001; see Table 1). The strength of this relationship supports the prediction that intergroup threat may be a driver of attitudes toward the outgroup.

#### Replication

We also collected feeling thermometers for these four generations in Studies 2 and 3, and replicated the attitudinal patterns described above (see Table 2; details in Supplementary Material).^3^ In all studies, Millennials felt significantly less favorably toward Baby Boomers than toward any other generation, and vice versa for Baby Boomers toward Millennials (all *p*s < .001; see Table 2).

**Table 2.** Millennials and Baby Boomers’s Attitudes Toward the Four U.S. Adult Generations, Studies 1–3.

Participant generation	Target generation	Study 1 *M* (*SD*)	Study 2 *M* (*SD*)	Study 3 *M* (*SD*)
Millennials	Millennials	6.9 (2.7)	6.9 (2.5)	6.7 (2.6)
	Generation-X	6.6 (2.4)	6.3 (2.2)	6.2 (2.2)
	Baby Boomers	5.6 (2.9)	5.3 (2.8)	5.0 (2.9)
	Silent Generation	6.4 (2.8)	6.4 (2.6)	6.3 (2.8)
Baby Boomers	Millennials	5.3 (2.8)	6.4 (2.8)	6.3 (2.5)
	Generation-X	6.6 (2.2)	7.3 (2.1)	7.1 (2.1)
	Baby Boomers	7.7 (2.1)	8.0 (2.2)	8.0 (2.1)
	Silent Generation	7.7 (2.5)	8.4 (1.9)	8.1 (2.2)

*Note.* Gray rows indicate the outgroup of interest (i.e., Millennials for Baby Boomer participants and Baby Boomers for Millennial participants). In all studies, Millennials felt significantly less favorably toward Baby Boomers than toward any other generation, and vice versa for Baby Boomers toward Millennials (all *p*s < .001).

## Study 2

In Study 1, Millennials and Baby Boomers reported more negative attitudes and higher levels of threat toward one another than toward other generations. The goal of Study 2 was twofold. First, we examined Millennials’s and Baby Boomers’s mutual animosity across a more varied set of outcome measures; second, we tested whether realistic and symbolic threats differentially predicted these various expressions of outgroup hostility.

### Methods

#### Participants

As per our preregistration, we sought to collect a sample equivalent in size to that of Study 1, but with a more equitable ratio of Millennial to Baby Boomer participants. We collected 401 complete responses on the crowdsourcing platform Prolific. After excluding duplicates, our sample was made of 184 Millennials (age: *M* = 31.08, *SD* = 4.62; 92 women; 29.3% ethnic minorities: 15 African Americans, 24 Asian Americans, 8 Latinos/Hispanic Americans, 1 Pacific Islander, 5 participants who identified as mixed race, and 1 participant who preferred not to state) and 200 Baby Boomers (age: *M* = 64.11, *SD* = 5.29; 122 women; 10.0% ethnic minorities: four African Americans, two Asian Americans, six Latinos/Hispanic Americans, three participants who identified as mixed race, four participants whose ethnic background was not listed, and one who preferred not to state). Including duplicate responses into the analyses did not materially change our conclusions.

#### Procedure

After a brief demographic questionnaire, participants completed a feeling thermometer about each generation. Participants were then told that they would be randomly assigned to an in-depth survey about one of the three outgroup generations. In fact, all Millennial participants evaluated Baby Boomers, and Baby Boomer participants, Millennials. We used this minor deception to mask the fact that our study focused on the Millennial-versus-Boomer tension specifically, thereby reducing risks of demand characteristics. A debrief concluded the study.

#### Measures

Full-scale measures are available in the Appendix.

##### Realistic and Symbolic Threats

Six realistic threat items (e.g., “[Baby Boomers/Millennials] get more from this country than they contribute”; α = .93) and six symbolic threat items (e.g., “[Baby Boomers/Millennials] have a different moral code than [Millennials/Baby Boomers]”; α = .88) were created specifically for this study and measured on a 7-point scale with endpoints 1 = *Strongly Disagree* and 7 = *Strongly Agree*.

##### Outgroup Stereotypes

Participants shared the extent to which they thought that eight stereotypes applied to outgroup members using a 7-point scale with endpoints 1 = *Not at All* and 7 = *A Great Deal* (e.g., burdensome, selfless (r), rude, respectful (r); α = .88). The items were selected for their high level of overlap between Baby Boomer and Millennial stereotypes (Cuddy & Fiske, 2002; Francioli & North, 2021b).

##### Outgroup Attitudes

Attitudes toward the outgroup were captured in two different ways. We used feeling thermometers similar to those of Study 1, and five items adapted from Danbold & Huo, 2022 (e.g., “I have a positive attitudes toward [Millennials/Baby Boomers]”), measured on a 7-point scale with endpoints 1 = *Strongly Disagree* and 7 = *Strongly Agree* (α = .91).

##### Policy Support

A series of six pro-young-adults policy items (e.g., “There should be full forgiveness of student debt”; “The AARP [American Association of Retired Persons] should do more to educate older adults about the values of younger generations”) and six pro-older-adults policy items (e.g., “More tax revenue should be reallocated to today’s seniors”; “Social media should be regulated to ensure that the voice of older adults can still be heard”) were created specifically for this study and utilized a 7-point scale with endpoints 1 = *Strongly Disagree* and 7 = *Strongly Agree*. An exploratory factor analysis did not reveal any obvious multifactorial structure for these 12 items, so we reverse-coded the six pro-older-adults items and combined them with the six pro-young items to form a single comprehensive policy measure (α = .69). To ease interpretation and simplify our analyses, we also reverse-coded the measure for Baby Boomers, so our final measure reflected participant endorsement of pro-ingroup policies for both Baby Boomers and Millennials.

### Results

Per our preregistered analytical plan, we ran separate multiple regressions for each DV (stereotyping, feeling thermometer, outgroup attitude scale, and pro-ingroup policies) with the following predictors: participant generation (binary: Baby Boomer = 0; Millennial = 1), realistic threat (continuous, standardized), symbolic threat (continuous, standardized), the interaction between generation and realistic threat, and the interaction between generation and symbolic threat. Per our preregistration form, eight participants were excluded because their score on either threat measure was ±2.5 *SD* away from the mean—analyses including outliers did not affect our overall findings. To ease interpretation and comparisons across dependent variables, we reverse-coded the thermometers and the attitude scale, such that higher scores represented more negative attitudes toward the outgroup. See Table 3 for detailed descriptive statistics and correlation matrix.

**Table 3.** Study 2 Descriptive Statistics and Correlation Matrix.

Participants	Measure	*M*	*SD*	α	(1)	(2)	(3)	(4)	(5)
Millennials	(1) Realistic threat	4.26	1.61	.93					
	(2) Symbolic threat	4.82	1.04	.80	.48				
	(3) Stereotyping	3.75	1.15	.84	.66	.45			
	(4) Attitudes (thermo)	5.32	2.82		−.47	−.33	−.61		
	(5) Attitudes (scale)	4.49	1.51	.92	−.61	−.43	−.84	.69	
	(6) Policies	4.28	0.88	.75	.53	.32	.63	−.58	−.65
Baby Boomers	(1) Realistic threat	2.98	1.37	.91					
	(2) Symbolic threat	4.40	1.31	.89	.65				
	(3) Stereotyping	4.11	1.09	.88	.54	.71			
	(4) Attitudes (thermo)	6.37	2.83		−.44	−.54	−.68		
	(5) Attitudes (scale)	4.87	1.23	.89	−.55	−.66	−.77	.77	
	(6) Policies	4.46	0.86	.74	.54	.56	.51	−.41	−.52

*Note.* All correlations significant at *p* <.001.

**Figure 2.**
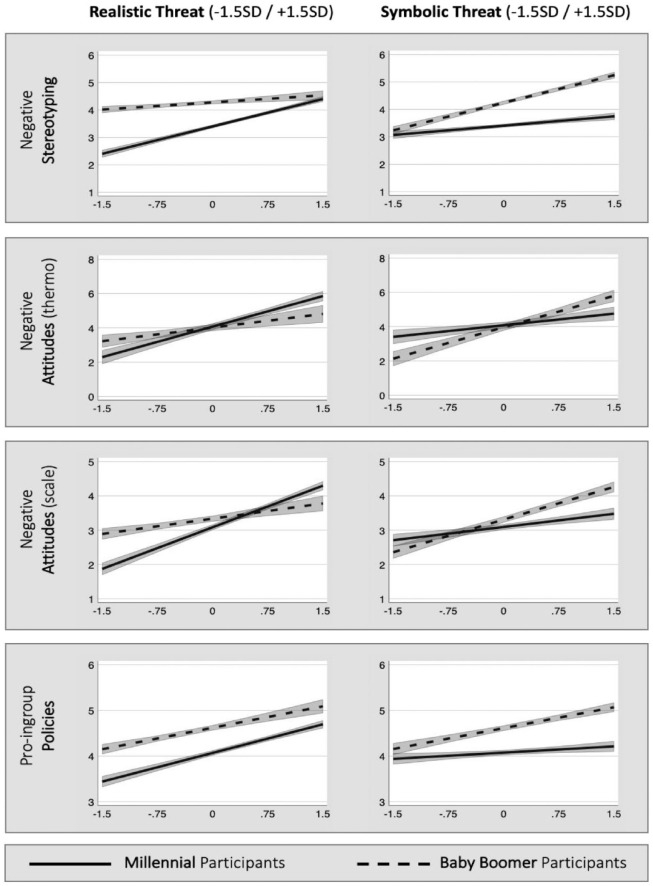
Relationship Between Threats and Outgroup-Relevant Outcomes Split by Participant Generation. *Note.* Steeper slopes for realistic threat among Millennials and symbolic threat among Baby Boomers reveal a threat asymmetry. Value of realistic threat at +1.5 *SD* = 6.03 and at −1.5 *SD* = 1.17; symbolic threat at +1.5 *SD* = 6.41 and at −1.5 *SD* = 2.80. Shaded areas indicate ±1 *SE*.

Regression outputs are in Table 4, interaction patterns, in Figure 2. As seen in the left panel of Figure 2, across all outcomes, realistic threat was a stronger predictor of outcome measures for Millennials (solid line) than Baby Boomers (dashed line). Conversely, and as seen in the right panel, symbolic threat was a stronger predictor of outcome measures for Baby Boomers (dashed line) than Millennials (solid line).

**Table 4.** Study 2 Outgroup Stereotyping, Outgroup Attitudes, and Pro-Ingroup Policy Support as a Function of Participant Generation, and Realistic and Symbolic Threats.

	Outgroup stereotypes				Attitudes (thermo, rev.)				Attitudes (scale, rev.)				Pro-ingroup policies			
Independent variables	*B*	*p*	95% CI	η_p_^2^	*B*	*p*	95% CI	η_p_^2^	*B*	*p*	95% CI	η_p_^2^	*B*	*p*	95% CI	η_p_^2^
Millennials	−0.84	<.001	[−1.02, −0.66]	.185	0.11	.683	[−0.43, 0.65]	.000	−0.22	.066	[−0.45, 0.01]	.009	−0.53	<.001	[−0.69, −0.37]	.102
Realistic Threat	0.18	.054	[−0.00, 0.36]	.130	0.54	.050	[−0.00, 1.08]	.062	0.30	.011	[0.07, 0.53]	.132	0.32	<.001	[0.16, 0.48]	.129
Millennials × Real. Thr.	0.50	<.001	[0.27, 0.72]	.049	0.62	.072	[−0.06, 1.30]	.009	0.51	.001	[0.22, 0.80]	.031	0.12	.244	[−0.08, 0.32]	.004
Symbolic Threat	0.68	<.001	[0.53, 0.83]	.153	1.23	<.001	[0.79, 1.68]	.065	0.64	<.001	[0.45, 0.83]	.106	0.31	<.001	[0.18, 0.44]	.041
Millennials × Symb. Thr.	−0.43	<.001	[−0.65, −0.20]	.037	−0.73	.033	[−1.40, −0.06]	.012	−0.30	.040	[−0.59, 0.01]	.011	−0.21	.036	[−0.41, −0.01]	.012
Constant	4.24	<.001			3.95	<.001			3.30	<.001			4.60	<.001		
*R* ^2^	.502	<.001	[0.43, 0.55]		.299	<.001	[0.22, 0.36]		.436	<.001	[0.36, 0.49]		.335	<.001	[0.253, 0.396]	

*Note.* Real. Thr. = Realistic Threat; and Symb. Thr. = Symbolic Threat. Millennials is a binary variable (0 = Baby Boomers; 1 = Millennials). Realistic threat and symbolic threat were standardized using grand means and standard deviations. Per preregistration script, responses including a realistic threat or symbolic threat score ±2.5 *SD* away from the grand mean were excluded from analyses (analyses including outliers did not materially change the results). CI = confidence interval.

Gray rows represent the primary predictors of interest.

#### Replication

We collected some of these same measures in Study 3 and tried to replicate the above findings for the attitudinal scale and thermometer (see details in Supplemental Material). Again, in the sample of Study 3—twice larger and better representative of the two generations’ gender, race, and political makeup—realistic threat was a stronger predictor of unfavorable attitudes toward the outgroup for Millennial participants (thermometer: *B* = 0.83, *p* < .001, η_p_^2^ = .015, 95% CI = [0.003, 0.035]; scale: *B* = 0.72, *p* < .001, η_p_^2^ = .056, 95% CI _=_ [0.030, 0.088]), and symbolic threat for Baby Boomer participants (thermometer: *B* = −0.44, *p* = .021, η_p_^2^ = .006, 95% CI _=_ [0.000, 0.020]; scale: *B* = −0.30, *p* < .001, η_p_^2^ = .014, 95% CI _=_ [0.003, 0.033]).

## Study 3

In Studies 1 and 2, Millennials and Baby Boomers exhibited negative biases toward one another across a wide range of measures. Furthermore, as predicted, in Study 2, realistic threat drove Millennials’s bias against Baby Boomers better than did symbolic threat, and symbolic threat drove Baby Boomers’s bias against Millennials better than did realistic threat.

In Study 3, we examined whether we might be able to alleviate these tensions. The threat asymmetry we uncovered in Studies 1 and 2 suggests that it might be difficult to develop a single threat intervention that directly caters to each generation’s unique concerns simultaneously. Furthermore, interventions that target outgroup threat directly can foster strong reactance (Rios et al., 2018). To bypass these issues, we developed an alternative informational intervention aimed at reducing perceived generational entitativity of generations and emphasizing instead the continuous, fluid, universal and more unifying experience of aging (e.g., the notion that today’s older adults are yesterday’s young, and today’s young, tomorrow’s old). Prior work has shown that reducing the perceived entitativity of a group can help alleviate intergroup conflict (Effron & Knowles, 2015). Furthermore, as our intervention targeted the entitativity of generations in general, rather than anything specific about either generation, we expected the material to be effective at reducing threat and prejudice for both Millennial and Baby Boomer participants.^4^

### Methods

#### Participants

We collected 1,057 complete responses from the U.S. participant pool of the crowdsourcing platform Prolific. We used strict screening criteria to build a participant sample more representative of Millennials’s and Baby Boomers’s demographic characteristics with regard to age, race, gender, and political orientation. After excluding respondents with failed attention checks or duplicate IP addresses, our final sample included 557 Millennials (Age: *M* = 31.7, *SD* = 4.4; 278 women; 42.4% ethnic minorities: 53 African Americans, 61 Asian Americans, 47 Latinos/Hispanic Americans, 8 Native Americans, 2 Pacific Islanders, 61 participants who identified as mixed race, 3 participants whose ethnic background was not listed, and 1 participant who declined to respond; political orientation: extremely liberal, 11.5%; liberal, 32.1%; moderate, 23.9%; conservative, 26.8%; and extremely conservative, 5.7%) and 366 Baby Boomers (age: *M* = 63.4, *SD* = 4.8; 213 women; 10.4% ethnic minorities: 15 African Americans, three Asian Americans, six Latinos/Hispanic Americans, two Native Americans, five participants who identified as mixed race, four participants whose ethnic background was not listed, and three participants who declined to respond; political orientation: extremely liberal, 10.7%; liberal, 34.4%; moderate, 16.9%; conservative, 29.5%; and extremely conservative, 8.5%).^5^ Including observations with IP duplicates or failed attention checks did not materially alter our results.

#### Procedure and Measures

Participants were introduced to our manipulation under the guise of a prestudy survey assessing social scientists’ effectiveness at conveying academic findings to a lay audience. Participants first read one of two mock newspaper interviews, which served as the control and the intervention condition.

**Figure 3.**
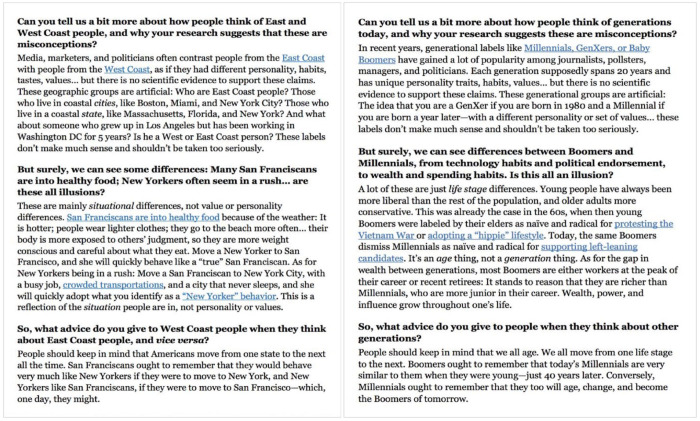
Study 3 materials. Control Condition on the Left and Intervention on the Right.

Both excerpts featured a purported interview with a social psychology professor at a top U.S.-based university. The intervention condition focused on generational identities, whereas the control condition emphasized regional ones (i.e., East Coasters and West Coasters). Each version included three questions from the interviewer, accompanied by the interviewee’s responses. Although the exact language of the two versions differed, the content matched in length and paralleled each other, albeit transposed to different contexts (i.e., temporal differences for the intervention and geographical differences for the control condition; see Figure 3). A pilot study (*N* = 79) pretested the material and confirmed the clarity, believability, and low reactance of both conditions (see Supplemental Material). To aid the credibility of our cover story, participants also answered three reading comprehension questions (e.g., “According to this researcher, [personality differences between East Coast and West Coast are not scientifically validated/generational labels such as Baby Boomers and Millennials are not scientifically valid].”). After completing the manipulation materials, participants were thanked and redirected to what was described as the “real” study they had been recruited for. The study started with a basic demographic questionnaire, followed by attitude thermometer items for each generation. Once again, participants were told that they would be randomly assigned to one of the three outgroup generation conditions, but Baby Boomers all evaluated Millennials, and Millennials all evaluated Baby Boomers. They did so by completing the outgroup attitude scale (α = .93), realistic threat measure (α = .95), and symbolic threat measure (α = .88) used in Study 2.

To assess potential demand characteristics, at the end of the study, we invited participants to explain what they thought the purpose of the study was. We had two research assistants independently code these text responses to identify participants who correctly guessed that the reading comprehension was an intervention meant to influence their attitudinal responses, κ = .84, *p* < .001 (98.8% agreement; disagreements settled by the first author).

### Results

See Table 5 for descriptive statistics and correlation matrix.

**Table 5.** Descriptive Statistics and Correlation Matrix, Study 3.

Participants	Measure	*M*	*SD*	α	(1)	(2)
Millennials	(1) Realistic threat	3.99	1.70	.95		
	(2) Symbolic threat	4.66	1.06	.81	.43	
	(3) Outgroup attitudes	4.56	1.46	.93	−.71	−.40
Baby Boomers	(1) Realistic threat	2.58	1.23	.92		
	(2) Symbolic threat	4.13	1.30	.89	.53	
	(3) Outgroup attitudes	5.29	1.13	.88	−.40	−.53

*Note.* Correlations all significant at *p* < .001.

As stated in our preregistered analytical plan, we excluded an additional 31 participants who scored +/−2.5 *SD* away from the mean on one of our key variables. Including these observations did not alter our conclusions. Per our analytical plan, we examined both the direct and indirect effects of our intervention on attitudes toward the outgroup generation to test its effectiveness. A regression analysis suggests that the total effect of the intervention increased positive outgroup attitudes, *B* = 0.20, *p* = .030, η^2^ = .005, 95% CI = [0.000, 0.019].

**Figure 4.**
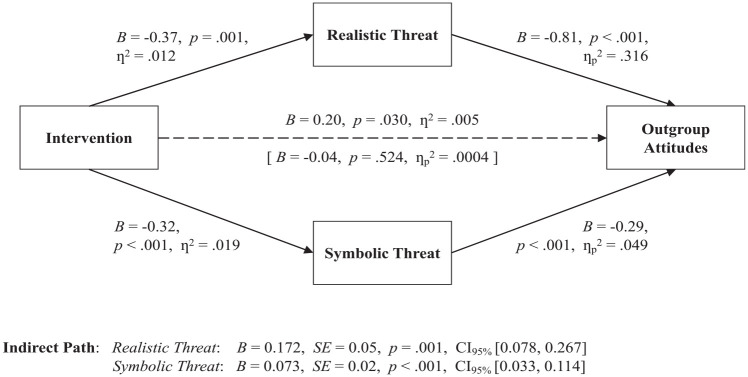
Mediational Process, Study 3. *Note.* Participants reported more positive outgroup attitudes in the intervention (vs. control) condition. This effect was mediated by both perceived realistic and perceived symbolic threats. CI = confidence interval.

Next, we used *sureg* and *nlcom* commands in Stata 15.1 (StataCorp, 2021) to perform a multiple-mediated model looking at the indirect effect of participant condition on attitudes toward the outgroup via both realistic and symbolic threats. The intervention significantly alleviated perceived realistic threat, *B* = -0.37, *p* = .001, η^2^ = .012, 95% CI _=_ [0.002, 0.030], and symbolic threat, *B* = -0.32, *p* < .001, η^2^ = .019, 95% CI = [0.005, 0.040]. Consistent with our preregistered predictions and our theorizing that the positive effect of the intervention would be mediated by realistic and symbolic threat, both indirect paths were significant: realistic threat, *B* = 0.17, *p* = .001, 95% CI _=_ [0.07; 0.28], and symbolic threat, *B* = 0.07, *p* < .001, 95% CI _=_ [0.03, 0.11] (see Figure 4).

#### Post Hoc Analyses

By challenging the legitimacy and entitativity of generational groups—rather than the validity of the unique intergroup threats experienced by each generation—we hoped that the intervention would be effective at alleviating the anxiety and hostility of both Millennials and Baby Boomers. Consistent with this goal, we did not find evidence that the effectiveness of the intervention was moderated by participant generation. For instance, a two-way ANOVA with the intervention and participant generation (Baby Boomer = 0; Millennial = 1) as predictors revealed no significant interactions for outgroup attitudes, *F*(1, 888) = 0.08, *p* = .778, η_p_^2^ = .0001, 95% CI _=_ [0.000, 0.005]; realistic threat, *F*(1, 888) = 0.11, *p* = .736, η_p_^2^ = .0001, 95% CI _=_ [0.000, 0.005]; or symbolic threat, *F*(1, 888) = 1.55, *p* = .214, η_p_^2^ = .002, 95% CI _=_ [0.000, 0.011]. In addition, we did not find evidence of three-way interactions in which the intervention reduces the effect of symbolic threat on outgroup attitudes for Baby Boomers more strongly than for Millennials, and the effect of realistic threat on outgroup attitudes for Millennials more strongly than for Baby Boomers. That said, it is worth acknowledging that we are likely underpowered to capture such a complex interaction model.

As a robustness check, we also examined participants’ descriptions of what they thought our study was about. We found little evidence that demand characteristics drove the effectiveness of our intervention. Only 40 participants (4.3%) correctly identified our manipulation in their essay. Excluding them strengthened the effect of our intervention on outgroup attitudes, *B* = 0.23, *p* = .014, η^2^ = .007, 95% CI _=_ [0.000, 0.022] and did not materially change the effect of the intervention on perceived realistic or symbolic threat. Although not definitive, these findings potentially alleviate concerns of demand characteristics and social desirability.

## General Discussion

Although scholars have challenged the empirical validity of popular generational categories, the social identities and narratives surrounding generations still inform the way many Americans make sense of their increasingly age-diverse world (Aboim & Vasconcelos, 2014; Costanza et al., 2012; National Academies of Sciences, Engineering, and Medicine, 2020; North & Shakeri, 2019; Parry & Urwin, 2011; Rudolph et al., 2021; Timonen & Conlon, 2015). In an exploratory survey, a preregistered correlational study, and a preregistered experiment, we built on ITT (Stephan et al., 2009) to examine the intergroup conflict between Baby Boomers and Millennials, today’s largest adult generations in the United States. We found that members of both generations express more hostile attitudes toward one another than toward other generations (Studies 1–3), a mutual animosity rooted in perceived intergroup threat (Studies 1–3). Consistent with our predictions about the asymmetrical nature of generational threats, the hostility of Baby Boomers (the dominant group) toward Millennials (the nondominant group) was driven primarily by symbolic threat, and the hostility of Millennials toward Baby Boomers by realistic threat (Studies 2–3). Finally, an intervention challenging the legitimacy and entitativity of generational categories successfully reduced perceived threat and hostile outgroup sentiments for members of both generations (Study 3).

### Theoretical Contributions

#### Contributions to Research on Intergenerational Conflict and Age-Based Social Cognition

This research advances social psychologists’ understanding of intergenerational relations by providing a theoretically grounded explanation for frequently observed antagonisms between America’s two largest proverbial birth cohorts. Although Millennials and Baby Boomers report reciprocal, acrimonious sentiments toward one another, the animosity of Millennials (i.e., the younger generation) is driven primarily by concerns over tangible resources (realistic threat) and the animosity of Baby Boomers (i.e., the older generation), by concerns over values and worldviews (symbolic threat). By identifying this asymmetric threat pattern, we provide social psychologists with a basic framework to make sense of contemporary intergenerational tensions. In addition, this framework showcases the value of studying biases targeting younger and older generations jointly. By examining both sides simultaneously, researchers can best contrast the degree and nature of hostilities targeting each of the two groups.

Our work also contributes to the literature on social cognition in the context of age, and more specifically, on perceptions of older and younger adults. Consistent with our findings, prior research has shown that younger people often disparage their elders (Nelson, 2005; North & Fiske, 2012) and that these older adults also disparage younger people (Bratt et al., 2020; Chasteen et al., 2021; Francioli & North, 2021b; Protzko & Schooler, 2019). However, when viewed through a generational lens, the nature of this disparagement is distinct. Our findings that Millennials view Baby Boomers as domineering and resource-withholding contrasts sharply with the “doddering but dear” stereotype commonly associated with older adults (Cuddy & Fiske, 2002). This observation suggests that generation-based social categories (e.g., Baby Boomers) may conjure social evaluations and stereotype contents distinct from those of more widely studied, age-based social categories (e.g., older adults). Although viewing older adults through the lens of age may trigger benevolence and paternalistic feelings (Glick & Fiske, 2001), viewing them generationally may elicit much more antagonistic attitudes. Future work may want to examine further what situational features trigger a benevolent (age-based) versus a more hostile (generation-based) social evaluation of older adults.

With regard to social evaluations of younger adults, prior social psychological work on age biases has largely focused on younger adults’ derogatory views of older generations (see North & Fiske, 2012 for a review) but less so on older adults’ impressions of—and attitudes toward—younger generations. Per our findings, the strength of Baby Boomers’s scorn toward Millennials stresses the potency of anti-young ageism. Bias against the young might hinder older generations’ generativity (i.e., “the concern for and commitment to the well-being of future generations,” McAdams & Logan, 2004). This eventuality is particularly concerning in a rapidly aging world where younger adults are facing major challenges that cannot be addressed without older generations’ cooperation (e.g., lower economic opportunities, climate change, and deficit of Social Security). This also underscores the need for more academic investigations on anti-young ageism, as recent psychological research has argued (Bratt et al., 2020; Chasteen et al., 2021; Francioli & North, 2021b).

#### Contributions to Intergroup Research

Our work also highlights how studying generational identities can advance our understanding of intergroup relations. Two examples stand out. First, prior research on intergroup threat and group status suggests that realistic threat tends to drive the negative outgroup sentiments of dominant groups toward nondominant groups (Morrison et al., 2009; Riek et al., 2006; Rios et al., 2018; Stephan et al., 2009). In contrast, we found consistent evidence that the animosity of Baby Boomers (economically and politically dominant) toward Millennials is primarily driven by symbolic threat. We encourage intergroup threat researchers to consider whether generations are unique in this regard—because of idiosyncratic features such as the ephemeral nature of generations or the dynamic nature of their societal standing—or whether there is something about intergenerational conflict that may generalize to other intergroup settings.

Second, our findings contribute to scholarship on group entitativity and intergroup bias. Prior work has shown that perceived entitativity can exacerbate intergroup biases (Agadullina & Lovakov, 2018; Campbell, 1958). However, more work is needed to determine whether challenging the legitimacy and coherence of group identities can effectively reduce threat and prejudice. Contrasting with prior work, which has focused specifically on reducing ingroup entitativity (Effron & Knowles, 2015), we find promising evidence that challenging both ingroup and outgroup entitativity simultaneously can reduce perceived threat and prejudice.

Given that researchers have long shown that, just like generations, race, and gender are socially constructed (e.g., Butler, 2004; Eagly, 2013; Jackman, 1994; Lorber & Farrell, 1991; Ridgeway, 2011; Sidanius & Pratto, 2001; West & Zimmerman, 1987), the effectiveness of our interventions suggests a potentially fruitful path toward reducing biases in these domains too. In this regard, of noteworthy mention, our intervention offers promising evidence that manipulating group entitativity can help reduce not only intergroup prejudice but also intergroup threat. However, while challenging the entitativity of generational categories, we also drew participants’ attention to age, a distinct but closely related alternate identity. Unlike generation, which is fixed and divisive, age is continuous, fluid, and universally experienced. Drawing participants’ attention to age may have, therefore, facilitated participants’ connections to outgroup members, reminding them that they have been or will be their age one day. Our ability to discredit generational identities was likely eased by this opportunity to substitute popular generational categories with a less divisive source of social identity. This observation highlights yet another notable aspect of studying age and generations.

### Limitations and Future Directions

This work has several limitations that future research can address. As noted at the outset, we focus on only two of the four—and soon to be five—proverbial adult generations in the United States. Whether future conflicts between Generation Z and Generation X come to mirror those between Millennials and Baby Boomers can tell us much about the cyclical nature of intergenerational tensions. Such a research investigation will help determine whether our findings reflect a recurrent relational pattern between younger and older generations, or whether these observed antagonisms are shaped mainly by the sum of idiosyncratic historical factors faced only by Millennials and Baby Boomers (e.g., Millennials coming of age through multiple recessions). Unfortunately, this question can only truly be answered with time.

Another set of limitations concerns our intervention study (Study 3). First, the informational material of our intervention combined multiple messages, including arguments about both the illegitimacy of generational identities and the transient and universal nature of age. Although we used this approach intentionally—to increase the odds of connecting with members of both generations—we acknowledge that combining multiple arguments makes it difficult to identify how each contributed to the effectiveness of the intervention. In the future, parsing these arguments could help identify a more precise mechanism at play and opportunities to improve the effectiveness of our intervention. Second, despite our best efforts to address the risks of demand characteristics and social desirability (see our Study 3 robustness checks), we cannot rule out entirely the possibility that these methodological artifacts might have inflated the effectiveness of our intervention. With this in mind, we invite readers to remain cautious when interpreting these findings and encourage independent replication.

Our work also focuses exclusively on the United States. Anecdotal evidence suggests that the United States may not be the only country dealing with intergenerational tensions and antagonistic generational narratives. For example, tensions between Millennials and Baby Boomers are a frequent topic of debate in the United Kingdom (The Guardian, 2022). In addition, in 2019, a Millennial politician in New Zealand went viral using the catchphrase “OK, Boomer” to shush a heckling Baby Boomer parliamentarian (The Guardian, 2019). Beyond these anecdotes of similar intergenerational dynamics outside the United States, we encourage scholars to examine how our findings may hold or vary across national and cultural contexts.

In addition, and as noted before, participants in our samples were predominantly White Americans (74% of our overall sample). We found no significant moderation of our primary effects when we contrasted responses from White and non-White Americans—nor did we find moderations by gender and level of education.^6^ That said, we were underpowered to run analyses disaggregating the ethnic minority groups in our sample. As such, our studies cannot speak conclusively to whether and how race may moderate intergenerational perceptions. We strongly encourage future researchers to explore this critical question and thereby provide a more comprehensive and nuanced picture of intergenerational relations in the United States.

Finally, our implementation of realistic threat revolved largely around economic issues regarding jobs, housing, and financial security. We hope that future work will also explore whether Millennials similarly perceive Baby Boomers to be a threat to their safety, health, and well-being, particularly as it relates to the perceived roles that older generations play in addressing issues such as climate change, pro-gun policies, and anti-abortion laws.

### Implications for Society

Popular narratives contrasting generations are commonplace in American society. While these claimed generational differences are rarely supported by data (National Academies of Sciences, Engineering, and Medicine, 2020), our findings suggest that generational identities are socially meaningful. We find that beliefs in the legitimacy of generational identities underlie animosity toward outgroup generations. In a context where wealth inequalities between younger and older adults have greatly increased (Pew Research Center, 2011), where major societal issues divide generations (e.g., climate change, abortion, and presidential elections; Pew Research Center, 2018, 2021b, 2022), and where important intergenerational challenges are arising (e.g., future insolvency of Social Security; Social Security Administration, 2004), popular narratives purporting to contrast artificial generational groups risks exacerbating the tensions we observed and weaken the potential for intergenerational solidarity.

Media professionals, corporate trainers, and consultants should, therefore, carefully weigh the costs of promoting these narratives. They may want to trade these generational tales for societal commentaries that focus on life stage differences, a more universal and scientifically valid analytical lens. In doing so, they may also want to acknowledge the situational factors leading certain age groups to behave differently today than they were in the past; this strategy may prove useful in reducing people’s natural tendency to inappropriately attribute outgroup’s predicaments to their own wrongdoing (e.g., today’s young do not struggle economically because they are a lazy generation, but rather, because of economic challenges they face that previous generations at the same age were spared of).

## Conclusion

By exploring the rift opposing Baby Boomers and Millennials, we show how tensions between the two largest adult generations in the United States are driven by asymmetrical experiences of threats. We also leverage the unique relationship between age and generational identities to reframe people’s thinking in a way that seems to reduce perceptions of threats and tensions. In doing so, we not only advance our understanding of contemporary intergenerational relations. We also unearth insights about intergroup conflicts more broadly, highlighting the benefits for social psychologists to further study intergenerational issues. We hope that this work inspires further research into this major societal rift and fosters more promising strategies to promote intergenerational harmony in a rapidly aging world.

## Supplemental Material

sj-docx-1-psp-10.1177_01461672231164203 – Supplemental material for Millennials Versus Boomers: An Asymmetric Pattern of Realistic and Symbolic Threats Drives Intergenerational Tensions in the United States Supplemental material, sj-docx-1-psp-10.1177_01461672231164203 for Millennials Versus Boomers: An Asymmetric Pattern of Realistic and Symbolic Threats Drives Intergenerational Tensions in the United States by Stéphane P. Francioli, Felix Danbold and Michael S. North in Personality and Social Psychology Bulletin

## Appendix

### Measures [Millennial Participant Version], Studies 2 and 3

Out-group Stereotype Measure (Study 2: α = .86)

Burdensome Selfish   Lazy     Rude

Generous (r) Selfless (r) Industrious (r) Respectful (r)

Out-group Attitudinal Measure (Study 2: α = .91; Study 3: α = .95)

I have a positive attitude toward [Baby Boomers].

I admire [Baby Boomers].

I enjoy being around [Baby Boomers].

I don’t like [Baby Boomers]. (r)

I hate [Baby Boomers]. (r)

Realistic Threat (Study 2: α = .93; Study 3: α = .89)

[Baby Boomers] get more from this country than they contribute.

[Baby Boomers] have more economic power than they deserve in this country.

[Baby Boomers] are preventing [Millennials] from achieving financial security.

[Baby Boomers] are draining the resources of [Millennials].

[Baby Boomers] take up more than their fair share of jobs.

[Baby Boomers] take up more than their fair share of the housing market.

Symbolic Threat (Study 2: α = .88; Study 3: α = .87)

[Millennials] and [Baby Boomers] have different family values.

The work ethic of [Baby Boomers] is at odds with the work ethic of [Millennials].

[Baby Boomers] have a different moral code than [Millennials].

[Baby Boomers]’ philosophy of life is in conflict with that of [Millennials].

The political views of [Baby Boomers] are incompatible with those of most Americans.

[Baby Boomers]’ approach to social interactions is not compatible with those of [Millennials].

Policy-oriented Measure (Study 2: α = .69)

There should be full forgiveness of student debt.

Older workers in age to retire should be encouraged to leave the workforce to free up jobs for younger generations.

More should be done to give Millennials access to affordable homeownership.

Baby Boomers should make more of an effort to understand and embrace the norms of younger generations.

The AARP (American Association of Retired Persons) should do more to educate older adults about the values of younger generations.

The media should do more to persuade Baby Boomers to adopt the beliefs of Millennials.

More tax revenue should be reallocated to today’s seniors. (r)

Workplaces should be encouraged to hire more older workers. (r)

More money should go into health care for today’s elderly. (r)

Social media should be regulated to ensure that the voice of older adults can still be heard. (r)

School programs should focus more on instilling traditional American values into young people. (r)

Mainstream media should do more to teach Millennials the morals of older generations. (r)

## Notes

1. Generation X (born 1965–1980) is numerically smaller and discussed less (half as much as Baby Boomers and a quarter as much as Millennials; Google Trends, 2021). The Silent Generation (1928–1945) is even smaller and talked about even less. Discussion about Generation *Z* (1997-undefined) is growing, but at the time of this writing, there is no consensus around the chronological endpoints of this generation (Pew Research Center, 2019). Furthermore, most Generation Z members are still in childhood—as young as 5 years, according to some proposed brackets. In the way that race and ethnicity researchers often focus on only two racial subgroups at a time (e.g., Black and White Americans), we opted to focus specifically on Baby Boomers and Millennials.

2. Given the exploratory nature of this study, we also collected responses from Gen-Xers. We describe the general pattern of their responses in Supplemental Material. Similarly, we included additional measures, listed in the Supplemental Materials, and included in the shared data files.

3. The replication in Study 3 is based on data collected after a manipulation. The results hold when we control for the manipulation or look at either condition in isolation.

4. Although we also considered a third condition testing whether enhancing the legitimacy of generational categories amplifies bias, the relatively small pool of Baby Boomers on crowdsourcing platforms precluded us from doing so with sufficient statistical power.

5. The smaller sample of Baby Boomers—relative to Millennials—is reflective of their underrepresentation in online data collection panels. Our efforts to recruit a politically representative sample of Baby Boomers have likely exhausted the available pool of Baby Boomer respondents on Prolific.

6. We compiled the thermometer responses of Studies 1 to 3 and ran a series of multilevel models to examine whether the “Millennials versus Boomers” antagonism varied across demographic groups (*N* = 1,714 participants * 4 target generations = 6,856 observations; 53.4% women; 26.0% non-White; average education = 12.4 years of formal education, *SD* = 1.8). Consistent with recent research on ageism (Francioli et al., 2022), we observed no significant moderation by gender, education, nor race.
