# Supplementary material for: Millennials Versus Boomers: An Asymmetric Pattern of Realistic and Symbolic Threats Drives Intergenerational Tensions in the United States
Source: Pers Soc Psychol Bull. 2023 May 3;50(11):1546–62. doi: 10.1177/01461672231164203 (PMC11490062; doi:10.1177/01461672231164203)
Supplement: sj-docx-1-psp-10.1177_01461672231164203 – Supplemental material for Millennials Versus Boomers: An Asymmetric Pattern of Realistic and Symbolic Threats Drives Intergenerational Tensions in the United States [file sj-docx-1-psp-10.1177_01461672231164203.docx]

**Millennials vs Boomers**

Supplementary Material

**Study 1 (Generation X participants)**

In a more exploratory way, we collected responses from GenXers as part of Study 1 (*N* = 178; Age: *M* = 44.0, *SD* = 4.45; 98 women; 30 non-White minorities). To examine their attitudes toward the four target generations (i.e., Millennials, Generation X, Baby Boomers, and Silent Generation), we ran a one-way repeated-measure ANOVA with target generation as predictors and attitudinal thermometer as the dependent variable, *F*(3, 531) = 55.45, *p* < .001. GenXers perceived their ingroup (*M* = 7.98 out of 10, *SD* = 1.90) the most positively, above Millennials (*M* = 4.98, *SD* = 2.77), Δ = 3.01, *p* < .001, CI_95%_ [2.51, 3.51], Baby Boomers (*M* = 6.55, *SD* = 2.82), Δ = 1.43, *p* < .001, CI_95%_ [0.93, 1.93], and Silents (*M* = 7.58, *SD* = 2.43), Δ = 0.40, *p* < .001, CI_95%_ [0.10, 0.90].

We then ran a one-way repeated-measure ANOVA with outgroup generations as predictors (Millennials/Baby Boomers/Silent Generation) and general threat as the dependent variable, *F*(2, 354) = 52.45, *p* < .001. GenXers reported feeling the highest level of threat from Millennials (*M* = 48.79 out of 100, *SD* = 35.01), above Baby Boomers (*M* = 33.21, *SD* = 33.22), Δ = 15.58, *p* < .001, CI_95%_ [9.57, 21.59], and Silents (*M* = 17.49, *SD* = 25.38), Δ = 31.30, *p* < .001, CI_95%_ [25.29, 37.31].

**Study 2 (replication of Study 1)**

A 2 (participant generation: Millennials versus Baby Boomer) x 4 (target generation: Millennials/GenXers/Boomers/Silents) mixed ANOVA on attitude thermometers revealed a significant two-way interaction, *F*(3, 1,122) = 44.77, *p* < .001. Largely consistent with the findings of Study 1, Millennials rated Boomers the least favorably (*M* = 5.32 out of 10, *SD* = 2.82), below GenXers (*M* = 6.27, *SD* = 2.20), Δ = -0.95, *p* < .001, CI_95%_ [-1.36, -0.54], Silents (*M* = 6.44, *SD* = 2.61), Δ = -1.12, *p* < .001, CI_95%_ [-1.53, -0.70], and ingroup members (*M* = 6.92, *SD* = 2.50), Δ = -1.60, *p* < .001, CI_95%_ [-2.01, -1.19]. Conversely, Boomers rated Millennials the least favorably (*M* = 6.37, *SD* = 2.83), below GenXers (*M* = 7.31, *SD* = 2.13), Δ = -0.94, *p* < .001, CI_95%_ [-1.34, -0.54], ingroup members (*M* = 7.98, *SD* = 2.16), Δ = -1.61, *p* < .001, CI_95%_ [-2.01, -1.21], and Silents (*M* = 8.39, *SD* = 1.88), Δ = -2.03, *p* < .001, CI_95%_ [-2.43, -1.63].

One-way, repeated measure ANOVAs with general threat as a dependent variable and outgroup generations as a predictor were significant for both Millennials, *F*(2, 362) = 33.84, *p* < .001, and Boomers, *F*(2, 386) = 70.99, *p* < .001. Consistent with Study 1, Millennials identified Boomers as the biggest threat to their generation (*M* = 42.62, *SD* = 32.12), above GenXers (*M* = 32.15, *SD* = 27.70), Δ = 10.47, *p* < .001, CI_95%_ [5.92, 15.02], and Silents (*M* = 23.62, *SD* = 26.33), Δ = 19.99, *p* < .001, CI_95%_ [14.45, 23.54]. Also consistent with Study 1, Boomers identified Millennials as the biggest threat to their generation (*M* = 34.29, *SD* = 34.45), above GenXers (*M* = 21.55, *SD* = 25.71), Δ = 12.74, *p* < .001, CI_95%_ [8.77, 16.73], and Silents (*M* = 10.18, *SD* = 19.44), Δ = 24.11, *p* < .001, CI_95%_ [20.13, 28.09]. These results were highly consistent with those of with Study 1.

**Pilot study to calibrate the experimental material of Study 3**

We conducted a pilot study to ensure that the material developed for the control and treatment conditions of Study 3 were comparable with regards to clarity, believability, and reactance. We collected 79 complete responses, 41 from Millennial participants and 38 from Baby Boomers (Age: *M* = 47.19, *SD* = 16.71; 39 women; 17.8% minorities).

A series of one-way ANOVAs were used to compare responses in the intervention versus control condition. Clarity was assessed using the reading comprehension questions used in Study 3. Participants in the control condition scored 99.2% (*SD* = 0.01) in comprehension, which did not significantly differ from those in the intervention condition, 98.3% (*SD* = 0.01), *F*(1, 79) = 0.37, *p* = .547. Reactance was captured using eight items measured on a 7-point scale with endpoints 1 = *Disagree Strongly* and 7 = *Agree Strongly* and inspired by the Salzburger state reactance scale (Sittenthaler et al., 2015; α = .91). Reactance was marginally higher in the control (*M* = 3.42, *SD* = 0.33) than in the intervention condition (*M* = 2.61, *SD* = 0.34) but remained overall low, *F*(1, 79) = 3.00, *p* = .087. Believability was captured using items assessing participants’ general level of agreement with the material on a scale of 1 = *Strongly Disagree* to 7 = *Strongly Agree* (see items and alphas below). Agreement was generally high, and participants in the control condition (*M* = 4.86, *SD* = 0.25) did not differ significantly from those in the intervention condition (*M* = 5.07, *SD* = 0.25), *F*(1, 79) = 0.36, *p* = .548.

**Study 3 (replication of Study 1,** attitudinal measure only**)**

We used the thermometers, attitude scale, and threat measures to replicate the findings of Study 1 and 2. Consistent with Study 1, a mixed 2-way ANOVA with attitude thermometers as a dependent variable showed a significant two-way interactions between participant generation (Millennial / Baby Boomer) and target generation (Millennials / Generation X / Baby Boomers / Silent Generation), *F*(3, 2,670) = 106.34, *p* < .001. Follow-up post-hoc tests confirmed that Boomers harbored less positive attitudes toward Millennials (*M* = 6.33, *SD* = 2.51) than toward GenXers (*M* = 7.11, *SD* = 2.13), Δ = -0.77, *p* < .001, CI_95%_ [-1.07, -0.47], Baby Boomers (*M* = 7.98, *SD* = 2.07), Δ = -1.65, *p* < .001, CI_95%_ [-1.95, -1.35], and Silents (*M* = 8.09, *SD* = 2.24), Δ = 1.75, *p* < .001, CI_95%_ [-2.05, -1.45]; and, conversely, Millennials reported less positive attitudes toward Boomers (*M* = 4.99, *SD* = 2.86) than toward their ingroup (*M* = 6.74, *SD* = 2.63), Δ = -1.75, *p* < .001, CI_95%_ [-1.99, -1.51], GenXers (*M* = 6.21, *SD* = 2.17), Δ = -1.59, *p* < .001, CI_95%_ [-1.47, -0.98], or members of the Silent Generation (*M* = 6.29, *SD* = 2.80), Δ = -1.30, *p* < .001, CI_95%_ [-1.55, -1.06]. These results were highly consistent with those of with Study 1.

**Study 3 (replication of Study 2,** attitudinal measures only**)**

To test whether realistic threat was more predictive of Millennials’ attitudes toward Boomers and symbolic threat of Boomers toward Millennials, we regressed participant generation (binary: Millennial = 1; Baby Boomer = 0), realistic threat (continuous, standardized), symbolic threat (continuous, standardized), the interaction between generation and realistic threat, and the interaction between generation and symbolic threat, on outgroup attitude, similar to Study 2. Both the attitude scale and thermometer served as dependent variables. Per the two interaction terms in our attitude *scale* model, realistic threat was a better predictor of attitudes for Millennial than for Baby Boomer participants, *B* = -0.72, *SE* = 0.10, *p* < .001, η_p_^2^ = .056, and symbolic threat, more predictive for Baby Boomer than Millennial participants, *B* = 0.30, *SE* = 0.08, *p* < .001, η_p_^2^ = .014.

Per the two interaction terms in our *thermometer* model, realistic threat was a better predictor of attitudes toward the outgroup generation for Millennial than Boomer participants, *B* = -0.83, *SE* = 0.23, *p* < .001, η_p_^2^ = .015, and symbolic threat for Boomer than Millennial participants, *B* = 0.44, *SE* = 0.19, *p* = .021, η_p_^2^ = .006. These results were highly consistent with the findings of with Study 2.

**Measures, Pilot Study for Study 3**

**Reactance** (α = .91)

***The researcher's statement made me feel...***

frustrated, upset, annoyed, disturbed

good, relieved, happy, positive (reverse coded items)

**Agreement with the material, Intervention Condition** (α = .91)

- Generational labels such as Baby Boomers and Millennials are artificial.
- Differences attributed to generations are mostly *life stage* effects.
- People should think of other generations as their past or future selves rather than as outgroups.
- Young Baby Boomers were probably very similar to Millennials today.
- As they get older, Millennials will probably be very similar to Baby Boomers.

**Agreement with the material, Control Condition** (α = .92)

- Geographic labels such as East Coast and West Coast are artificial.
- Differences attributed to geographic attributes are mostly *situational* effects.
- People should keep in mind that they would quickly adopt local behaviors if they were to move to a new city or state.
- East Coast people would probably behave very similarly to West Coast people if they lived on the West Coast.
- West Coast people would probably behave very similarly to East Coast people if they lived on the East Coast.

**Experimental Manipulation, Study 3**

*Control Condition*

**Please read the following excerpt from a recent interview of James F. Winfried, a Professor of social psychology at New York University and an expert on intergenerational study.**

**Can you tell us a bit more about how people think of East and West Coast people, and why your research suggests that these are misconceptions?**

Media, marketers, and politicians often contrast people from the East Coast with people from the West Coast, as if they had different personality, habits, tastes, values... but there is no scientific evidence to support these claims. These geographic groups are artificial: Who are East Coast people? Those who live in coastal *cities*, like Boston, Miami, and New York City? Those who live in a coastal *state*, like Massachusetts, Florida, and New York? And what about someone who grew up in Los Angeles but has been working in Washington DC for 5 years? Is he a West or East Coast person? These labels don’t make much sense and shouldn’t be taken too seriously.

**But surely, we can see some differences: Many San Franciscans are into healthy food; New Yorkers often seem in a rush… are these all illusions?**

These are mainly *situational* differences, not value or personality differences. San Franciscans are into healthy food because of the weather: It is hotter; people wear lighter clothes; they go to the beach more often… their body is more exposed to others’ judgment, so they are more weight conscious and careful about what they eat. Move a New Yorker to San Francisco, and she will quickly behave like a “true” San Franciscan. As for New Yorkers being in a rush: Move a San Franciscan to New York City, with a busy job, crowded transportations, and a city that never sleeps, and she will quickly adopt what you identify as a “New Yorker” behavior. This is a reflection of the *situation* people are in, not personality or values.

**So, what advice do you give to West Coast people when they think about East Coast people, and *vice versa*?**

People should keep in mind that Americans move from one state to the next all the time. San Franciscans ought to remember that they would behave very much like New Yorkers if they were to move to New York, and New Yorkers like San Franciscans, if they were to move to San Francisco—which, one day, they might.

**Reading Comprehension items**

***Please respond to the following true or false statements. According to this researcher…***

…personality differences between East Coast and West Coast are not scientifically validated.

…differences between San Franciscans and New Yorkers are largely a reflection of situational factors.

…if a New Yorker was to move to San Francisco, he or she would quickly behave like a San Franciscan.

*Intervention Condition*

**Please read the following excerpt from a recent interview of James F. Winfried, a Professor of social psychology at New York University and an expert on intercultural study.**

**Can you tell us a bit more about how people think of generations today, and why your research suggests these are misconceptions?**

In recent years, generational labels like Millennials, GenXers, or Baby Boomers have gained a lot of popularity among journalists, pollsters, managers, and politicians. Each generation supposedly spans 20 years and has unique personality traits, habits, values... but there is no scientific evidence to support these claims. These generational groups are artificial: The idea that you are a GenXer if you are born in 1980 and a Millennial if you are born a year later—with a different personality or set of values… these labels don’t make much sense and shouldn’t be taken too seriously.

**But surely, we can see differences between Boomers and Millennials, from technology habits and political endorsement to wealth and spending habits. Is this all an illusion?**

A lot of these are just *life stage* differences. Young people have always been more liberal than the rest of the population, and older adults more conservative. This was already the case in the 60s, when then young Boomers were labeled by their elders as naïve and radical for protesting the Vietnam War or adopting a “hippie” lifestyle. Today, the same Boomers dismiss Millennials as naïve and radical for supporting left-leaning candidates. It’s an *age* thing, not a *generation* thing. As for the gap in wealth between generations, most Boomers are either workers at the peak of their career or recent retirees: It stands to reason that they are richer than Millennials, who are more junior in their career. Wealth, power, and influence grow throughout one’s life.

**So, what advice do you give to people when they think about other generations?**

People should keep in mind that we all age. We all move from one life stage to the next. Boomers ought to remember that today’s Millennials are very similar to them when they were young—just 40 years later. Conversely, Millennials ought to remember that they too will age, change, and become the Boomers of tomorrow.

**Reading Comprehension items**

***Please respond to the following true or false statements. According to this researcher…***

…generational labels such as Baby Boomers and Millennials are not scientifically valid.

…differences attributed to generations are mostly life stage effects.

…people should think of other generations as their past or future selves rather than outgroups.

**Additional Measures, Study 1-3**

Study 1 Demographics (born in the U.S.; Level of education; Political view), Current Standing, Future Standing, Current Prototypicality, Future Prototypicality, Ingroup and Outgroup Stereotyping, Outgroup Differences, Realistic Threat, Prototypicality Threat, Outgroup Assimilation, Sense of Generational Belonging.

Study 2 Demographics (Level of education; Political view), Prototypicality Threat.

Study 3 Demographics (Political view), Reading Comprehension items.

**Supplementary Material, Reference**

Sittenthaler, S., Traut-Mattausch, E., Steindl, C., & Jonas, E. (2015). Salzburger state reactance scale (SSR scale). *Zeitschrift für Psychologie*.
